# Depressive symptomatology in the first-episode schizophrenia spectrum disorders OPTiMiSE trial: prevalence, correlates, symptom progression and outcomes

**DOI:** 10.1038/s41537-025-00681-3

**Published:** 2025-11-13

**Authors:** Javier-David Lopez-Morinigo, David Fraguas, Covadonga M. Diaz-Caneja, Joaquin Galvañ, Gregor Berger, Stefan Leucht, Inge Winter-van Rossum, Celso Arango, Carmen Moreno

**Affiliations:** 1 Department of Child and Adolescent Psychiatry, Institute of Psychiatry and Mental Health, Hospital General Universitario Gregorio Marañón, IiSGM, School of Medicine, Universidad Complutense. Centro de Investigación Biomédica en Red de Salud Mental (CIBERSAM), Madrid, Spain; 2 “Centro” Mental Health Center, Institute of Psychiatry and Mental Health, IdISSC, Hospital Clínico San Carlos, Madrid, Spain; 3 Department of Child and Adolescent Psychiatry and Psychotherapy, University Hospital of Psychiatry Zurich, Zurich, Switzerland; 4 Department of Psychiatry and Psychotherapy, Technical University Munich, Munich, Germany; 5 Department of Psychiatry, UMC Utrecht Brain Center, University Medical Center Utrecht, Utrecht, The Netherlands

**Keywords:** Psychosis, Schizophrenia

## Abstract

Numerous aspects of depressive symptomatology in first-episode schizophrenia spectrum disorders (FES) remain unclear. Based on data from the FES OPTiMiSE trial, we estimated the prevalence of depression (Calgary Depression Scale for Schizophrenia (CDSS) total score ≥7) at baseline (*n* = 122, 27.5%) and at weeks 4 (*n* = 57, 15.9%) and 10 (*n* = 14, 21.5%). Baseline depression was cross-sectionally associated with more severe extrapyramidal symptoms (*p* = 0.036) and poorer subjective wellbeing (*p* < 0.001). At week 4, baseline depression was linked to poorer psychosocial functioning (*p* < 0.001) and subjective wellbeing (*p* < 0.001). At week 10, baseline depression was associated with psychosis non-remission (*p* = 0.042) and worse subjective wellbeing (*p* = 0.011). There was a significant correlation between decrease in CDSS and PANSS total scores (*p* < 0.001) at weeks 4 and 10. Depressive symptomatology in the FES OPTiMiSE trial was prevalent and associated with poorer objectively- and subjectively-measured outcomes over a 10-week follow-up. Early intervention for depression may improve outcomes in FES.

## Introduction

Schizophrenia has been conceptualised as *non-affective* psychosis since the first Kraepelinian dichotomous classification^1,2^, on which the international classifications of mental disorders have been based^3^. However, both manic and depressive symptoms appear to be intrinsic to schizophrenia^4,5^ and the continuum model of psychoses has been well-supported by genetics^3^, neuroimaging^6^ and empirical^7^ studies.

Over two decades ago, the prevalence of depression in schizophrenia was estimated to range from 7% to 75%^8^. Such a wide range was probably due to relevant between-study differences in the definition of depression and the role of population-related social, environmental and cultural determinants of mental health^9^. To address this issue, the Calgary Depression Rating Scale for Schizophrenia (CDSS) was designed and validated^10^ and has been widely used ever since^11^. A 2020 meta-analysis of 53 CDSS-based observational studies estimated the prevalence of depression in schizophrenia at 28.6%, being the risk of depression higher in older schizophrenia patients^12^. Consistent with this, a later meta-analysis of 18 studies revealed 32.6% of schizophrenia outpatients to fulfill criteria for comorbid major depressive disorder^13^.

Although age—older age, higher risk of depression—was consistently found to be a major predictor of depression in schizophrenia patients^14^, other classic risk factors for depression in the general population, such as gender, yielded inconclusive results. One of the first studies on gender and depression in schizophrenia reported no gender differences in the prevalence of depression^15^. While a 2020 meta-analysis found male patients to more likely suffer from comorbid depression^12^, this finding was not replicated by a 2021 meta-analysis which revealed no between gender differences in the prevalence of depression in schizophrenia^13^. These discrepancies may be, in part, explained by some delay in diagnosis in females and gender differences in the course of the illness, with females showing a better overall outcome^16^.

More importantly, depressive symptoms in first-episode schizophrenia spectrum disorder (FES) were associated with negative outcomes, including treatment discontinuation^17^, psychosis non-remission^18^, increased risk of suicidal behaviour^19^, and poorer functioning^4,20–22^. Also, depressive symptomatology in schizophrenia is known to be clinically heterogenous^23^, encompassing different symptom trajectories over the course of psychosis. Overall depressive symptom severity tends to decrease in parallel with improvement in psychotic symptoms, particularly over the early stages of treatment^24^. While this may lead to speculation that depressive symptoms in schizophrenia may be amenable to treatment, previous evidence confirming this is still limited^25–27^ and no differentiating clinical characteristics between depressive symptom trajectories were identified^24^. Nevertheless, early depressive symptoms in schizophrenia patients may contribute to longer-term poorer outcome^4,20–22^, although this area remains under-researched.

Over one third of acutely psychotic patients develop Post-Psychotic Depression (PPD)^28^, although its nosological validity requires deeper theoretical debate^29^. PPD appears to occur independently of positive or negative symptom progression^30^. Rather, patients’ self-appraisal of psychosis and perceived social identity were found to underlie PPD^24,31,32^.

This noted, the true prevalence of depression in FES patients before and after treatment onset, its sociodemographic and clinical correlates, symptom progression and associations with longitudinal clinical and social outcomes remain unclear.

To fill this gap, we leveraged data from the Optimization of Treatment and Management of Schizophrenia in Europe (OPTiMiSE) project with a fourfold aim: (i) to estimate the prevalence of depression in FES; (ii) to investigate correlates of baseline depression in FES; (iii) to explore the depressive symptom progression over the trial period, and (iv) to examine the associations of baseline depression with five predetermined outcomes at 4- and at 10-week follow-up: psychosis remission, treatment discontinuation, illness severity, psychosocial functioning and subjective wellbeing. Based on the previous literature, we hypothesised: (i) that ~30% of participants will have depression at baseline, although less than half of them will remain depressed at four weeks after treatment onset; (ii) that having depression will be associated with older age; (iii) that depressive symptoms will evolve in parallel to psychotic symptoms, and (iv) that baseline depression will be associated longitudinally with lower rates of remission, higher rates of treatment discontinuation, greater illness severity, and worse psychosocial functioning and subjective wellbeing.

## Results

At baseline, *N* = 443 FES patients met the OPTiMiSE study inclusion criteria (Intention-to-Treat sample) and had CDSS data available. Of these, *n* = 371 completed Phase 1 (83.7%), of whom *n* = 358 had CDSS data available at that point (week 4).

Among Phase 1 completers with CDSS data available (*n* = 358), *n* = 245 individuals (68.4%) were in remission at the end of Phase 1 (week 4) and those who were not in remission (*n* = 113, 31.6%) were invited to initiate Phase 2. Of them, *n* = 85 (75.2%) started Phase 2 and *n* = 68 completed it, of whom *n* = 64 had CDSS ratings at the end of Phase 2 (week 10). Among Phase 2 completers with CDSS data (*n* = 64), *n* = 34 subjects (53.1%) were not in remission at week 10. For further details of the participant flowchart, see Fig. S1 in the online supplementary material.

### Prevalence of depression in the OPTiMiSE trial sample

At baseline, the mean CDSS total score was 4.55 ± 4.58, with *n* = 122 (27.5%) presenting with depression (CDSS total score ≥7) and only *n* = 103 subjects (23.2%) with a CDSS total score of 0—i.e., free of depressive symptoms -.

At week 4, CDSS total score mean was 2.83 ± 3.88, with *n* = 57 subjects (15.9%) meeting the CDSS threshold (≥7) for depression.

At week 10, CDSS total score mean was 3.92 ± 4.09, with *n* = 14 participants (21.5%) scoring 7 or above. Further details of the prevalence of individual symptoms (CDSS items) are provided in Table S1 in the online supplementary material.

### Group comparisons between patients with/without baseline depression

Bivariate analyses at baseline (Table 1) revealed that depressed individuals (compared with non-depressed ones), were more likely to be living alone (*p* = 0.018), had a longer duration of untreated psychosis (DUP) (*p* = 0.001), more severe extrapyramidal symptoms (EPS) (*p* = 0.001), were less likely in remission (*p* = 0.048), and showed greater illness severity (*p* = 0.005), and worse psychosocial functioning (*p* = 0.019) and subjective wellbeing (*p* < 0.001).

**Table 1:** Baseline characteristics of the whole sample and groups comparisons between patients with/without depression.

	Sample *N* = 443	Depression *n* = 122	Non-depression *N* = 321	Statistic	*p*-value
*Sociodemographic*					
Age (years)	25.0 ± 6.0	24.9 ± 5.9	25.6 ± 6.0	*t* = –1.07	0.28
Sex (females) (*n*, %)	132 (29.8)	42 (34.4)	90 (28.0)	*X*^2^ = 1.72	0.19
Unemployed	259 (58.5)	74 (60.6)	185 (57.6)	*X*^2^ = 0.33	0.56
Ethnicity (white)	381 (86.0)	102 (83.6)	279 (86.9)	*X*^2^ = 0.80	0.37
Living alone	82 (18.5)	31 (25.8)	51 (15.9)	*X*^2^ = 5.64	**0.018**
Unmarried	414 (93.4)	117 (95.9)	297 (92.5)	*X*^2^ = 1.65	0.20
Education (years)	12.3 ± 2.9	11.9 ± 3.3	12.4 ± 2.8	*t* = –1.65	0.099
*Clinical*					
Diagnosis (SZ)	225 (50.8)	66 (54.1)	159 (49.5)	*X*^2^ = 0.74	0.39
DUP (months, median)	4.00	6.00	3.00	U	**0.001**
UKU_EPS	0.87 ± 2.07	1.51 ± 2.57	0.65 ± 1.81	*t* = 3.29	**0.001**
*Psychopathology*					
PANSS_TOTAL	77.7 ± 18.8	84.9 ± 16.8	74.9 ± 18.7	*t* = 5.16	**<0.001**
PANSS_POSITIVE	12.4 ± 13.0	13.2 ± 3.9	12.1 ± 3.9	*t* = 2.81	**0.005**
PANSS_NEGATIVE	15.7 ± 6.6	16.4 ± 5.9	15.5 ± 6.7	*t* = 1.25	0.21
PANSS_DISORG.	8.6 ± 3.1	8.3 ± 2.9	8.6 ± 3.2	*t* = –0.98	0.33
PANSS_MANIA	7.6 ± 3.3	8.2 ± 3.4	7.3 ± 3.2	*t* = 2.59	0.010
PANSS_DEPRESSION	7.9 ± 3.3	11.2 ± 2.7	6.7 ± 2.6	*t* = 16.58	**<0.001**
Remission status	46 (10.4)	7 (5.7)	39 (12.1)	*X*^2^ = 3.91	**0.048**
*Illness severity*					
CGI total score	4.5 ± 0.9	4.7 ± 0.8	4.4 ± 0.9	*t* = 2.85	**0.005**
*Functioning*					
PSP total score	48.7 ± 15.5	45.8 ± 13.9	49.7 ± 15.9	*t* = –2.35	**0.019**
SWN total score	81.5 ± 16.0	69.0 ± 12.9	87.9 ± 14.6	*t* = –10.69	**<0.001**

*CDSS* Calgary depression scale for schizophrenia^10^, *SZ* schizophrenia, *DUP* duration of untreated psychosis, *AD* antidepressants, *CPZ* chlorpromazine, *UKU* Udvalg for Kliniske Undersogelser (UKU) Side Effect Rating Scale^78^, *EPS* extrapyramidal symptoms, *PANSS* positive and negative syndrome scale for schizophrenia^74^, *CGI* clinical global impression^75^, *PSP* personal and social performance scale^76^, *SWN* subjective wellbeing under neuroleptics^77^.

Bold values indicates statistically significant results (*p* < 0.05).

In the multivariable logistic regression model, only more severe EPS (odds ratio (OR) = 1.18, 95% CI 1.011–1.37, *p* = 0.036) and poorer subjective wellbeing (OR = 0.93, 95% CI 0.91–0.95, *p* < 0.001) were significantly associated with the odds of baseline depression (Table 2).

**Table 2:** Multivariable logistic regression model on baseline depression (CDSS ≥ 7).

	B	SE	Wald	*p*	Exp (B)	95% CI
Living alone	0.251	0.350	0.513	0.474	1.285	0.647–2.550
DUP	–0.006	0.023	0.066	0.797	0.994	0.950–1.040
**UKU_EPS**	**0.163**	**0.078**	**4.396**	**0.036**	**1.177**	**1.011–1.370**
PANSS_T	0.016	0.010	2.507	0.113	1.016	0.996–1.036
CGI	–0.278	0.213	1.709	0.191	0.757	0.499–1.149
PSP	–0.002	0.010	0.55	0.815	0.998	0.978–1.018
**SWN**	**–0.076**	**0.011**	**48.341**	**<0.001**	**0.926**	**0.907–0.947**

*CDSS* Calgary depression scale for schizophrenia^10^, *DUP* duration of untreated psychosis, *UKU* Udvalg for Kliniske Undersogelser (UKU) Side Effect Rating Scale^78^, *EPS* extrapyramidal symptoms, *PANSS_T: PANSS* positive and negative syndrome scale for schizophrenia^74^ total score. *CGI* clinical global impression^75^, *PSP* personal and social performance scale^76^, *SWN* subjective wellbeing under neuroleptics^77^.

Bold values indicates statistically significant results (*p* < 0.05).

### Correlations between depressive and psychotic symptom severity changes

There was a significant (*p* < 0.001) correlation between decrease in CDSS and PANSS total scores from baseline to week 4 (*r* = 0.32) and from week 4 to week 10 (*r* = 0.51).

From baseline to week 4 there was a significant correlation between change in depressive and negative psychotic symptom severity (*r* = 0.20, *p* < 0.001), which was also replicated from week 4 to week 10 (*r* = 0.26, *p* = 0.039). There was a significant correlation between change in depressive and positive psychotic symptom severity from week 4 to week 10 (*r* = 0.27, *p* = 0.037), but not from baseline to week 4 (see Table 3 for details).

**Table 3:** Bivariate correlations between changes in depressive and psychotic symptom severity.

	Depressive symptoms (CDSS total score)					
	Phase 1			Phase 2		
	*n*	*r*	*p*	*n*	r	*p*
PANSS_POSITIVE	342	0.023	0.678	**61**	**0.267**	**0.037**
PANSS_NEGATIVE	**358**	**0.197**	**<0.001**	**64**	**0.259**	**0.039**
PANSS_DISORGANISATION	358	0.055	0.302	64	0.199	0.115
PANSS_MANIA	358	0.095	0.074	64	0.134	0.290
PANSS_DEPRESSION	**358**	**0.617**	**<0.001**	**64**	**0.647**	**<0.001**
PANSS_TOTAL	**358**	**0.321**	**<0.001**	**64**	**0.514**	**<0.001**

*CDSS* Calgary depression scale for schizophrenia^10^, *PANSS* positive and negative syndrome scale for schizophrenia^74^.

Bold values indicates statistically significant results (*p* < 0.05).

### Relationship between baseline depression and longitudinal outcomes

There were no significant differences in psychosis remission rates at week 4 between participants with and without depression at baseline (63.0% vs 68.9%, respectively, *p* = 0.284). Participants with depression at baseline were less likely to achieve psychosis remission at week 10 than those without baseline depression (20.0% vs 43.3%, *p* = 0.019). The effect of baseline depression on psychosis remission at week 10 remained significant in the logistic regression model (OR = 0.33, 95%CI 0.11–0.98, *p* = 0.042), but not when conducting sensitivity analyses without those individuals already in remission at baseline (*n* = 46).

Baseline depression was significantly (*p* < 0.001) linked to greater illness severity and poorer psychosocial functioning and subjective wellbeing at week 4, but only to poorer subjective wellbeing (*p* = 0.011) at week 10 (Table 4).

**Table 4:** Relationship between baseline depression (CDSS ≥ 7) and continuous outcomes.

	End of Phase 1 Week 4		End of Phase 2 Week 10	
	Depressed	Non-depressed	Depressed	Non-depressed
*CGI*	3.5 ± 0.9	3.2 ± 1.1***	5.0 ± 0.7	5.0 ± 1.0
*PSP*	57.9 ± 16.1	63.3 ± 15.9***	45.9 ± 13.6	52.6 ± 19.5
*SWN*	80.9 ± 15.9	92.8 ± 14.3***	72.2 ± 16.6	86.4 ± 18.3*

Depressed: CDSS total score ≥7. Non-depressed: CDSS total score <7.

*CDSS* Calgary depression scale for schizophrenia^10^, *CGI* clinical global impression^75^, *PSP* personal and social performance scale^76^, *SWN* subjective wellbeing under neuroleptics^77^.

*p < 0.05; **p < 0.01, ***p < 0.001.

## Discussion

### Main findings

We analysed the OPTiMiSE trial dataset to investigate various aspects of depression in FES, including its prevalence, correlates, symptom progression and associations with longitudinal outcomes. Four main conclusions can be drawn from results. First, over one quarter of participants (27.5%) exhibited depression at baseline and 15.9% continued to do so after four weeks of treatment, which was consistent with hypothesis i. Second, more severe baseline EPS and poorer subjective wellbeing were associated with higher odds of baseline depression, although we had postulated older age to correlate with more severe depression (hypothesis ii). Third, depressive symptoms evolved in parallel with positive and negative psychotic symptoms from baseline to week 4 and from week 4 to week 10, supporting hypothesis iii. Finally, baseline depression was linked to non-remission at week 10 and greater illness severity and worse psychosocial functioning and subjective wellbeing at week 4 and at week 10, in line with our fourth hypothesis.

### Comparison to previous literature

At baseline, over one quarter of participants in the FES OPTiMiSE study (27.5%) had clinically meaningful depressive symptoms, defined as a CDSS total score equal to or above 7^33^, and only 23.3% of participants were free of depressive symptoms (i.e., CDSS total score =0). These results were in agreement with previous literature, including the aforementioned EUFEST trial, which reported 38.3% of participants to be depressed at baseline by using a lower CDSS threshold (≥6)^17^, and two recent meta-analyses yielding a pooled prevalence of depression in schizophrenia between 28.6%^12^ and 32.6%^13^. Based on a lower CDSS cut-off point (≥5), which was reported to be valid in an European sample^11^, the prevalence of depression in a previous OPTIMISE-based study with an minimally overlapping sample was estimated at 47.6^18^. Somehow conflicting with this, a study with schizophrenia patients in remission from India revealed a prevalence of depression of 27.6% (CDSS ≥ 5) and 18.8% (CDSS ≥ 7)^34^. Higher CDSS cut-off points, such as CDSS ≥ 6^35^ and ≥7^32^, were also considered in first-episode psychosis (FEP), which yielded a prevalence of depression of 15.1%^35^ and 59%^32^. Hence, the prevalence of depression in patients with schizophrenia spectrum disorders seems to depend on the illness phase and the threshold definition for depression.

In routine clinical practice, however, depressive symptoms among patients with schizophrenia spectrum disorders may have been largely under-recognised^4^ due to their challenging differential diagnosis from negative symptoms^8,36,37^, especially when not using specific instruments such as the CDSS. Interestingly, in children and adolescents presenting with first-episode early-onset psychosis, mania seems to behave as the most predominant and stable affective symptom dimension^38^, whereas depression may become more relevant in the non-acute phase of the illness^39^.

Only EPS and subjective wellbeing were significantly associated with baseline depression in the multivariable regression model. Owing to the OPTiMiSE inclusion/exclusion criteria detailed above, EPS among participants could not be attributed to previous exposure to antipsychotics. This may lead to speculation that depression and EPS in FES may be, to some degree, “two sides of the same coin”, which mirrors previous observations in Parkinson’s Disease^40^. Certainly, future research may build on this notion, that is, the relationship between extrapyramidal and depressive symptoms in FES and in Parkinson’s Disease.

Subjective wellbeing, a core patient-reported outcome measure, partially overlaps with adherence, recovery and quality of life^41^. However, no causality conclusions can be drawn from the cross-sectional associations between subjective wellbeing and depression at baseline, both of which may have an impact on each other, yet a research gap and an unmet clinical need in psychosis^42,43^.

Of relevance, we failed to replicate well-known demographic risk factors for depression in schizophrenia, such as (older) age^12,13^ and male sex^12^. This being said, our negative result concerning sex differences was consistent with some previous studies^13,15,16,44^.

This may have been due to the baseline characteristics of the sample, namely the high predominance of males across the board and the relatively narrow age range of a FES sample (interquartile range: 21–29 years). In other words, correlates of depression may differ between FES and non-FES patients. Alternatively, depression in psychosis and depression in the general population may behave as two different, albeit related, phenomena, which warrants deeper theoretical debate and more empirical research^4^.

To complicate matters further, depression in psychosis may have both trait- and state-like properties^32^. Thus, in the OPTiMiSE trial depressive symptoms evolved in parallel to positive and negative psychotic symptoms over Phase 1 and Phase 2, consistent with previous studies^17,30,45^. On the other hand, depressive symptoms might exhibit trait-like characteristics starting from the prodromal stage^32^, presenting a potential avenue for psychosis prevention by targeting these symptoms. Although the predictive value of depressive symptoms for transitioning to psychosis is not yet fully determined^46^, the Diagnostic and Statistical Manual of Mental Disorders, Fifth Edition (DSM-5) attenuated psychosis syndrome has been meta-analytically associated with comorbid depression^47^. Future clinical trials should therefore explore ways to optimise improvement in negative and depressive symptoms^48^, which can be, to some degree, delineated from each other when using validated instruments, such as the Positive and Negative Syndrome Scale for Schizophrenia (PANSS) and CDSS, respectively^49^.

In particular, in our cohort depressive symptom severity decreased as psychosis evolved towards remission, which replicated previous studies^24^. This said, no causality conclusions can be reached from the above associations between change in psychotic and depressive symptom severity. Moreover, previous meta-analyses of clinical trials testing the effect of antidepressants^25,26^ and antipsychotics^50^ on mood in patients with schizophrenia yielded modest overall results. This progression of both psychotic and depressive symptoms may be, in large part, explained by amisulpride, which was received by all Phase 1 participants and ~50% of Phase 2 participants. Indeed, amisulpride was demonstrated to be an efficacious antipsychotic^51^ with antidepressant properties in patients with schizophrenia spectrum disorders^52–54^.

So-called PPD was first described by Mayer–Gröss in 1920 as “future rejection and despair as a way of reacting after a psychotic experience”^28^. PPD has been demonstrated to be highly prevalent and a different nosological entity from antipsychotics side-effects and negative symptoms^29^, although evidence-based treatments for PPD in FEP are still lacking^55^.

Baseline depression in the FES OPTiMiSE trial was linked to poorer outcomes, namely non-remission at week 10 (but not at week 4), greater illness severity and worse psychosocial functioning at week 4 and poorer subjective wellbeing at week 4 and at week 10. In this regard, it should be noted that high remission rates were achieved by participants in the OPTiMiSE trial at the end of Phase 1 and Phase 2^56,57^. This may largely reflect the well-demonstrated efficacy of amisulpride, which was received by all participants in Phase 1 and 1 in 2 of Phase 2 participants, and olanzapine (50% of Phase 2 participants)^51^. In addition, the duration of the above trial phases, the study design and the definition of remission^58^ were likely to contribute to this finding. In keeping with this, baseline depression was linked to lower remission rates at week 10 (but not at week 4), which replicated a previous analysis with an overlapping sample^18^. This finding, however, may have been due to non-responders representing a subgroup of more severely ill patients, hence more depressed over the early course of psychosis. Consistent with our findings, previous meta-analyses linked depression in schizophrenia to poorer functional outcome^21^, social functioning^59^ and quality of life^60^, which largely overlaps with subjective wellbeing^42^, as measured in our study. Although meta-analytic evidence of the impact of depression on outcomes in FEP is still limited^61,62^, our results may suggest that early intervention on depression during a FES may contribute to recovery, which requires future clinical trials. Alternatively, meeting criteria for depression at first presentation with psychosis may simply identify a subgroup of more severely ill FES patients who will achieve poorer outcomes irrespective of depression-alleviating treatment.

### Strengths and limitations

Thanks to the OPTiMiSE project methodology, we could follow a relatively large sample of FES patients over a 10-week period, during which depressive symptoms severity and several clinically relevant outcomes were assessed with validated instruments, such as the CDSS, at various timepoints. As noted above, the CDSS threshold for depression (≥7) was higher than in some previous studies, thus reducing the risk of false positives.

However, certain limitations should be considered when interpreting the study results. First, the methodology of the OPTiMiSE trial, which was not originally designed to test the specific hypotheses of this study. As a result, some groups comparisons detailed above may have been underpowered. Second, all participants gave consent to a lengthy clinical trial protocol and these findings may not apply to real-world conditions. Also, only non-remission participants at week 4 were invited to start OPTiMiSE Phase 2, which may have affected the generalisability of results. In line with this, data from the OPTiMiSE Phase 3 were not considered due to two reasons. First, the very small size of Phase 3 subsample (*n* = 18) was found to be underpowered for group comparisons between patients with/without baseline depression. Second, only those who had not achieved remission at the end of Phase 1 and at the end of Phase 2 could initiate Phase 3, which would therefore have introduced a selection bias. Moreover, out of *n* = 40 non-remitters at the end of Phase 2 (*n* = 40), only *n* = 28 subjects agreed to start Phase 3, which was completed by *n* = 18. Third, further putative contributors to depression in schizophrenia, such as quality of life or loneliness, were not evaluated in this study. Data on antidepressants use were collected. However, the low number of participants on antidepressants at baseline (*n* = 46) led to insufficient power to detect significant differences between antidepressants users/non-users, which was also outside the scope of this study. Also, the efficacy of antidepressants in schizophrenia remains to be confirmed^25,26^. Specifically, future clinical trials should test whether antidepressants may influence the course of the psychotic illness and outcome. Fourth, depression was dichotomised and defined as a CDSS total score ≥7, which, although the best cut-off point according to the validation study of the scale^33^, may be arbitrary to some extent. In keeping with this, we built a logistic regression model with a restrictive significance level (at *p* < 0.05) for the inclusion of the independent variables, i.e., putative correlates of depression. Although the ‘capitalization on chance’ phenomenon can never be fully ruled out^63^, this seems unlikely given our hypothesis-driven approach and the fact that no model modifications were made. This said, some caution should be taken when considering the generalisability of the model^63,64^. Fifth, beyond the 10-week follow-up most outcomes could not be assessed due to attrition issues, including the long-term occurrence of depression in FES, its correlates, symptom progression and outcomes, hence a topic for further research.

### Implications on clinical practice and directions for future research

Overall, depression in schizophrenia spectrum disorders, especially in FES, remains under-researched^4^. In particular, evidence supporting treatments for depression in psychosis is limited. While two previous meta-analyses failed to show antidepressants augmentation to be superior to placebo in terms of efficacy^25^ and effectiveness^26^, second-generation antipsychotics (except ziprasidone) were meta-analytically demonstrated to reduce depressive and negative symptoms severity^50^. Future randomised controlled trials may test the efficacy of pharmacological treatments for the management of depression in schizophrenia, including newly-developed agents, such as lurasidone^65^, brexpiprazole^66^, cariprazine^67^, FDA-approved lumateperone^68^ or the muscarinic receptor agonist Xanomeline-Trospium (KarXT)^69^. In addition, adjunct psychotherapies, such as cognitive-behavioural therapy for psychosis^70^ and metacognitive training^71^, may contribute to boosting mood.

In summary, our study contributes to the growing body of research on depression in schizophrenia, yet an under-researched topic^4^. By using data from a well-designed FES clinical trial, namely the OPTiMiSE project, we replicated the relatively high prevalence of depressive symptomatology over the early course of the psychotic illness. More importantly, we showed the onset of depression to have a negative impact on objectively- and subjectively-measured outcomes over a 10-week follow-up. Routine clinical assessment and monitoring of depressive symptoms in FES should therefore be encouraged, for which validated instruments such as the CDSS should be used. Depression may thus become a treatment target in its own right within early intervention services. Certainly, early intervention of depressive symptoms may contribute to better long-term outcomes, including enhanced subjective wellbeing and quality of life, although this requires future large-scale clinical trials testing depression-alleviating interventions over more prolonged follow-up periods.

## Methods

### Sample, study design and procedure

Data for this study came from the 3-phase 27-center OPTiMiSE study, which was carried out across 14 European countries and Israel. The full methodology of the OPTiMiSE trial, which is registered at ClinicalTrials.gov (NCT01248195), was detailed elsewhere^56,57^. Briefly, inclusion criteria were: (i) age: 18–40 years; (ii) DSM-IV diagnosis - schizophrenia, schizophreniform disorder or schizoaffective disorder – confirmed with the Mini-International Neuropsychiatric Interview plus^72^; (iii) time period from the psychosis onset to the study inception shorter than 2 years; and (iv) previous use of antipsychotics for no more than 2 weeks in the preceding year or for less than 6 weeks lifetime. Other non-antipsychotic drugs were permitted as prescribed by the treating consultant. Participants gave written informed consent as approved by the local research ethics committees.

For the purposes of this study, we used OPTiMiSE Phase 1 and Phase 2 data. In Phase 1, participants were treated with amisulpride (200–800 mg/day) over 4 weeks in an open-label design. Those who did not achieve symptomatic remission^58^ after 4 weeks were randomly assigned to remain on amisulpride or to switch to olanzapine (5–20 mg/day) during a 6-week double-blind period.

### Depressive symptomatology assessment

Depressive symptoms severity was assessed with the Calgary Depression Scale for Schizophrenia (CDSS)^10^. The CDSS is comprised of 9 items enquiring about symptoms of depression, such as hopelessness, depressed mood, or guilty ideas. Each item is scored within a Likert scale ranging from 0 (symptom absent) to 3 (severe), which are summed up to create total scores ranging from 0 to 27—with higher scores reflecting more severe depression. The CDSS was meta-analytically found to have excellent internal consistency (0.83) and inter-rater reliability (0.88)^73^. Depression was defined as a CDSS total score ≥7 since a score above 6 was reported to have a specificity of 82% and a sensitivity of 85% for the presence of a major depressive episode^33^. Specifically, we estimated the prevalence of depression at baseline (Aim 1), we investigated the putative correlates of baseline depression (Aim 2), and we performed bivariate correlations between change in CDSS total scores and change in PANSS scores (see below) in Phase 1 and Phase 2 (Aim 3).

### Outcome measures

As outlined above (Aim 4) we investigated the associations between baseline depression and five predefined outcomes of interest—psychosis remission, treatment discontinuation, illness severity, psychosocial functioning and subjective wellbeing—at the end of Phase 1 (week 4) and Phase 2 (week 10).

*Symptomatic remission*—the primary outcome of the OPTiMiSE study^56,57^—was defined according to the criteria set out by Andreasen^58^, that is, a maximum rating of 3 on all the following items of the PANSS^74^: P1, P2, P3, N1, N4, N6, G5 and G9. Although originally a 6-month period of remission was required^58^, this criterion was not applied to the OPTiMiSE project^56,57^.

*Illness severity* was evaluated by means of the Clinical Global Impression (CGI) scale, i.e., higher scores indicate greater illness severity^75^.

*Treatment discontinuation* rates were estimated as the proportion of participants starting, but not completing, each treatment phase (i.e., the proportion of *non-completers*).

*Psychosocial functioning* was objectively measured with the Personal and Social Performance Scale (PSP), which is a clinician-based assessment of personal and social functioning^76^. Higher PSP total scores indicate better functioning.

We used the Subjective Wellbeing under Neuroleptics Scale (SWN)^77^ as a self-report measure of *subjective wellbeing*. The SWN is a 20-item scale, which includes 10 positive and 10 negative statements about oneself wellbeing, each of which is self-rated within a 1–6 Likert scale based on the degree of agreement. Individual items scores can be summed up to provide total scores, with higher scores reflecting better subjective wellbeing. The SWN has shown to have good performance in terms of validity and reliability^41^.

### Additional variables

Baseline sociodemographic variables included age, sex, employment, living, and marital status, and years of education. We also collected data on clinical diagnosis (schizophrenia vs. other psychoses), DUP and EPS as measured by a subscore from the neurological subscore from the Udvalg for Kliniske Undersogelser (UKU) Side Effect Rating Scale^78^. In particular, for this study purposes the sum of these six UKU items scores—Dystonia, Rigidity, Hypokinesia/Akinesia, Hyperkinesia, Tremor and Akhatisia—was taken as the EPS-UKU subscore. Since each UKU item is scored on a 0–3 scale, with higher scores indicating greater symptom severity, the EPS-UKU subscore ranged from 0 to 18.

### Statistical analysis

First, we estimated the crude prevalence of depression (CDSS total scores ≥7)^33^ at baseline, at week 4 and at week 10, including the corresponding 95% Confidence Intervals (CIs) (Aim 1). Second, a multivariable logistic regression model on odds of having depression at baseline as dependent variable was built, which included those significant variables (at *p* < 0.05) from group comparisons analyses between patients with/without depression (Aim 2). We decided to set the significance level for putative correlates of depression to be added to the multivariable logistic regression model at *p* < 0.05 in order to build a confirmatory model aimed at testing the study hypotheses, thus avoiding overfitting by excluding weakly associated variables (for instance, at *p* < 0.10). Third, we ran bivariate correlation analyses between change in CDSS total score and change in PANSS subscores and total score over Phase 1 and Phase 2 using non-parametric tests (i.e., Spearman coefficients) due to the skewed distribution of CDSS total score (Aim 3). Finally, we investigated the relationship between baseline depression and several predetermined outcomes at weeks 4 and 10 as follows (Aim 4). For binary outcome measures, logistic regression analyses on the odds of achieving psychosis remission and discontinuing treatment were performed, whereas three univariate analyses of the variance compared patients with/without baseline depression on each continuous outcome variable (i.e., CGI, PSP, and SWN total scores), whilst controlling for baseline data (ANCOVAs). Specifically, those significant variables from the bivariate analyses were taken as covariates, namely living status, DUP, EPS-UKU subscore and PANSS subscores.

It is fair to say that General Linear Mixed Models (GLMM) were reported to be particularly useful in longitudinal analyses of between-group differences by modelling fixed and random effects. Based on previous recommendations^79^ and given the limited power of relatively small subgroups and normal distribution of the outcome variables (i.e., CGI, PSP and SWN total scores), however, we decided to perform ANCOVA analyses. While Generalised Linear Mixed Models (GLMMs) may offer greater flexibility than ANCOVA, GLMMs appear to be less suitable for testing the relationship between baseline and longitudinal variables since GLMMs cannot be automatically adjusted for baseline (pre-treatment) differences in outcome variables. More specifically, ANCOVA was specifically designed to adjust for baseline variables (covariates), thus providing a more robust analysis, whilst controlling for this confounding effect^80^.

A significance level of 5% was set for the all the above analyses, which were performed with The Statistical Package for Social Science version 25.0 (SPSS, IBM Corp.; Armonk, NY, USA).

## Supplementary information

Table S1. Prevalence of depressive symptoms and depression at baseline, at week 4 and at week 10

Figure S1. Participant Flow Chart

## Data Availability

Data supporting these results are available upon reasonable request to the corresponding author, provided dataset access policy is complied with.
