# Supplementary material for: Depressive symptomatology in the first-episode schizophrenia spectrum disorders OPTiMiSE trial: prevalence, correlates, symptom progression and outcomes
Source: Schizophrenia (Heidelb). 2025 Nov 13;11(1):135. doi: 10.1038/s41537-025-00681-3 (PMC12615624; doi:10.1038/s41537-025-00681-3)
Supplement: Supplementary file 1 — Table S1. Prevalence of depressive symptoms and depression at baseline, at week 4 and at week 10 [file 41537_2025_681_MOESM1_ESM.doc]

| **Table S1. Prevalence of depressive symptoms and depression at baseline, at week 4 and at week 10** | | | | | | |
| --- | --- | --- | --- | --- | --- | --- |
| **CDSS items (present)** | |  | **BASELINE**  **N=443** | | **Phase 1**  **Week 4**  **n=358** | **Phase 2**  **Week 10**  **n=65** |
| 1. *Depressed mood* | | n (%) | 263 (59.4) | | 143 (39.9) | 37 (56.9) |
|  | | 95% CI | 54.8 – 64.0 | | 34.8 – 45.0 | 45.0 - 65.0 |
| 1. *Hopelessness* | | n (%) | 193 (43.6) | | 127 (35.5) | 29 (44.6) |
|  | | 95% CI | 38.9 – 48.2 | | 30.5 – 40.5 | 32.0 – 57.0 |
| 1. *Self-Depreciation* | | n (%) | 165 (37.2) | | 98 (27.4) | 26 (40.0) |
|  | | 95% CI | 32.7 – 41.8 | | 22.7 – 32.0 | 28.0 - 52.0 |
| 1. *Guilty ideas* | | n (%) | 132 (29.8) | | 52 (14.5) | 11 (16.9) |
|  | | 95% CI | 25.5 – 34.1 | | 10.9 – 18.2 | 8.0 – 26.0 |
| 1. *Pathological guilt* | | n (%) | 137 (30.9) | | 56 (15.6) | 14 (21.5) |
|  | | 95% CI | 26.6 – 35.2 | | 11.9 – 19.4 | 11.0 – 32.0 |
| 1. *Morning depression* | | n (%) | 121 (27.23) | | 73 (20.4) | 15 (22.1) |
|  | | 95% CI | 23.1 – 31.5 | | 16.2 – 24.5 | 13.0 – 34.0 |
| 1. *Early wakening* | | n (%) | 121 (27.3) | | 57 (16.2) | 9 (13.8) |
|  | | 95% CI | 23.1 – 31.5 | | 12.9 – 20.6 | 4.0 – 21.0 |
| 1. *Suicide* | | n (%) | 108 (24.4) | | 38 (10.9) | 12 (18.5) |
|  | | 95% CI | 20.4 – 28.4 | | 7.6 – 14.1 | 9.0 - 28.0 |
| 1. *Observed depression* | | n (%) | 182 (41.1) | | 110 (30.7) | 27 (41.5) |
|  | | 95% CI | 36.5 – 45.7 | | 25.9 – 35.5 | 29.0 – 54.0 |
| **CDSS TOTAL SCORE** | Mean ± SD | | | 4.55 ± 4.58 | 2.83 ± 3.88 | 3.92 ± 4.09 |
|  | | 95% CI | 4.12 - 4.98 | | 2.43 - 3.24 | 2.91 – 4.94 |
| **Prevalence of depression**  (CDSS total score ≥ 7) | | n (%)  95% CI | 122 (27.5)  23.4 – 31.7 | | 57 (15.9)  12.1 – 19.7 | 14 (21.5)  11.3 – 31.8 |

CDSS: Calgary Depression Scale for Schizophrenia.

Items were deemed “present” for individual scores ≥ 1.
