# Supplementary material for: Depressive symptomatology in the first-episode schizophrenia spectrum disorders OPTiMiSE trial: prevalence, correlates, symptom progression and outcomes
Source: Schizophrenia (Heidelb). 2025 Nov 13;11(1):135. doi: 10.1038/s41537-025-00681-3 (PMC12615624; doi:10.1038/s41537-025-00681-3)
Supplement: Supplementary file 2 — Figure S1. Participant Flow Chart [file 41537_2025_681_MOESM2_ESM.doc]

**N=446**

**Phase 1**

**Week 4**

CDSS unavailable, n=3

**CDSS**

**N=443**

**Enrolled and started Phase 1**

**Completed Phase 1**

**N=371**

**(CDSS, n=358)**

**Completed Phase 2**

**N=72**

**(CDSS, n=65)**

**Phase 2**

**Week 10**

Remission

n=250

non-remission

n=121

non-remission

n=40

Remission

n=32

Started Phase 2

n=93
